# Molecular dynamics simulation and bioinformatics study on chloroplast stromal ridge complex from rice (*Oryza sativa* L.)

**DOI:** 10.1186/s12859-016-0877-0

**Published:** 2016-01-12

**Authors:** Yubo Zhang, Yi Ding

**Affiliations:** State Key Laboratory of Hybrid Rice, Department of Genetics, College of Life Sciences, Wuhan University, Wuhan, 430072 People’s Republic of China

**Keywords:** Rice (*Oryza sativa* L.), Chloroplast stromal ridge complex, *Os*PsaC and *Os*PsaD, Molecular dynamics simulation, Bioinformatics study

## Abstract

**Background:**

Rice (*Oryza sativa* L.) is one of the most important cereal crops in the world and its yield is closely related to the photosynthesis efficiency. The chloroplast stromal ridge complex consisting of PsaC-PsaD-PsaE plays an important role in plant photosynthesis, which has been a subject of many studies. Till now, the recognition mechanism between PsaC and PsaD in rice is still not fully understood.

**Results:**

Here, we present the interaction features of *Os*PsaC and *Os*PsaD by molecular dynamics simulations and bioinformatics. Firstly, we identified interacting residues in the *Os*PsaC-*Os*PsaD complex during simulations. Significantly, important hydrogen bonds were observed in residue pairs R19-E103, D47-K62, R53-E63, Y81-R20, Y81-R61 and L26-V105. Free energy calculations suggested two salt bridges R19-E103 and D47-K62 were essential to maintain the *Os*PsaC-*Os*PsaD interaction. Supportively, electrostatic potentials surfaces of *Os*PsaD exhibited electrostatic attraction helped to stabilize the residue pairs R19-E103 and D47-K62. In particular, the importance of R19 was further verified by two 500 ns CG-MD simulations. Secondly, this study compared the stromal ridge complex in rice with that in other organisms. Notably, alignments of amino acids showed these two salt bridges R19-E103 and D47-K62 also existed in other organisms. Electrostatic potentials surfaces and X-ray structural analysis strongly suggested the stromal ridge complex in other organisms adopted a similar and general recognition mechanism.

**Conclusions:**

These results together provided structure basis and dynamics behavior to understand recognition and assembly of the stromal ridge complex in rice.

**Electronic supplementary material:**

The online version of this article (doi:10.1186/s12859-016-0877-0) contains supplementary material, which is available to authorized users.

## Background

Rice (*Oryza sativa* L.) is one of the most important cereal crops in Southeast Asia and sixty percentage of the population regard rice as the staple food in China. Improving photosynthesis efficiency can greatly increase rice yield. Photosynthesis occurs in the chloroplast and it begins when the electron is raised to a higher energy in a special chlorophyll molecule P680 of the photosystem II (PSII). Afterwards, the energy is transferred by an electron transport chain and four thylakoid membrane protein complexes are involved in the electron transferring. These complexes are photosystem I (PSI), PSII, cytochrome b_6_f and ATP-synthase complexes. PsaC, PsaD and PsaE of PSI are termed as “stromal ridge complex” as they locate in the stromal side of the thylakoid membrane. The main function of the stromal ridge complex is to dock electron acceptors ferredoxin and flavodoxin by PsaC binding to electron acceptors F_A_ and F_B_ [1].

Genetic deletion experiments [2–4] showed PsaC firstly bound to the PsaA/B heterodimer, and then PsaD bound to the prerequisite PsaA/B-PsaC complex, and finally PsaE bound to the PsaA/B-PsaC-PsaD complex. Supportively, X-ray crystallography [5, 6] exhibited PsaC, PsaD and PsaE located on the top of PsaA/B, containing a pseudo C2-symmetry [7]. This spatial conformation allows PsaC rotate around pseudo C2 symmetry by its binding to PsaA/B [7]. As the second assembly stromal ridge subunit, PsaD is supposed to bind the correctly bound-region of PsaC, while undock incorrectly-bound PsaC. Additionally, functional experiments [8, 9] showed only if PsaD bound to stromal ridge proteins, the electron acceptors F_A_ and F_B_ were able to maintain their final magnetic properties [8] and to reduce the ferredoxin [9].

These experimental observations together stress the importance of the PsaC-PsaD interaction in the formation of stromal ridge complex. However, the dynamics behavior of the PsaC-PsaD interaction is still obscure. Currently, the *in silico* studies are good complementary to experimental approaches. All-atomic (AT) and coarse grained (CG) molecular dynamics (MD) simulations can reflect protein dynamic behaviors in detail such as conformational change. Free energy calculation had been used to explore molecular mechanism [10] and drug scanning [11]. For instance, Zhang et al. [12] computed the free energy profile of lipid PS6 along its dissociation path of aquaporin V by a non-equilibrium molecular dynamics method, with the Brownian dynamics fluctuation dissipation theorem. This method was also used to determine the free energy profile of O_2_ along its permeation path, helping scientists to understand the dissociation constant and binding mechanics. Zhang et al. [10] employed computational alanine scanning based on the molecular mechanics generalized Born surface area (MM-GBSA) approach to identify one complex model pSOD-*Os*Tom20. Collectively, MD simulations combined with free energy calculations were efficient approaches to explore molecular binding mechanism in the protein complex.

In this study, we explored the recognition mechanism between *Os*PsaC and *Os*PsaD (*Os: Oryza sativa*) by MD simulations and bioinformatics analysis. By using MD simulations, important H-bonding interactions were identified. Free energy calculations suggested two salt bridges R19-E103 and D47-K62 were essential to maintain the interaction between *Os*PsaC and *Os*PsaD. Supportively, electrostatic potentials calculations and CG-MD simulations exhibited the importance of these two salt bridges. In addition, a bioinformatics analysis showed generality of these two salt bridges in other organisms.

## Methods

### Generation of atomic models of OsPsaCD complex

The modeling process was performed as our previous descriptions [10, 13, 14]. The amino acid sequence of the PsaC and PsaD (*Os*PsaC and *Os*PsaD) from *Oryza sativa* were obtained from the NCBI (ID: gi: 11466848 and gi: 115477831). In the first homology modeling step, template structures related to the PsaC and PsaD proteins were searched against the whole Protein Data Bank [15](PDB) using the Blast algorithm. We modeled complex structure *Os*PsaC-*Os*PsaD from *Oryza sativa* using the crystal structure from *Pisum sativum* (PDB id: 2WSC) as a template through the SWISS-MODEL [16].

In this work, residue numbering in the *Os*PsaD starts from the 66^th^ residue because the first 65 residues of the PsaD are not in the recognition region. This means that the first residue of the *Os*PsaD (Q1) in the main text is actually referred to as Q66.

### MD simulation with explicit solvent

The MD simulations were performed with the GROMACS 4.5.3 software package [17] using the OPLS force field [18] and the SPC216 water model. The protonation state of ionizable groups was chosen to correspond to pH 7.0. Counterions were added to compensate the net charge of the system. The initial structure of the complex was immersed in a periodic water box. The electrostatic interactions were calculated by using the Particle-mesh Ewald (PME) algorithm [19], and the van der Waals forces were treated with a cutoff distance of 10 Å. After 3000 steps of energy minimization using a steepest descent method, the system was subject to 300 ps of equilibration at 300 K and normal pressure, using harmonic position restraints with a force constant of 1000 kJ mol^−1^ nm^−2^. The system was coupled to an external bath by the Berendsen pressure and temperature coupling method [20]. The production run was performed under the same conditions except that all position restraints were removed. The results were analyzed using the standard software tools provided by the GROMACS package [17]. Calculation of free energy landscapes was performed with in-house scripts [10, 12, 13, 21, 22]. Visualization and manipulation of the conformations was performed using the programs visual molecular dynamics [23] and Swiss-PdbViewer 3.7 [16]. The distance analysis of the nonpolar residue-pairs was performed using the R statistical software package [24].

### Calculation of binding free energies by MM-GBSA

The binding free energy of the *Os*PsaCD was estimated by the MM-GBSA approach [25, 26]. In this study, the implicit generalized Born solvation model was used (igb = 2). The temperature was set to 300 K. Non-bonded interactions were cut off at a distance of 12 Å. The ff99 force field (Parm99) [27] was applied throughout the energy minimization and MD simulations.

In the MM-GBSA implementation of Amber 11.0 [28], the binding free energy of A + B → AB is calculated using the following thermodynamic cycle:
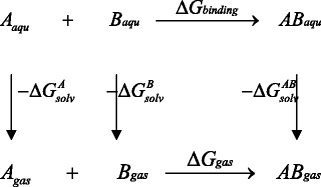

$$ \begin{array}{l}\varDelta Gbinding=\varDelta Ggas-\varDelta {G}_{solv}^A-\varDelta {G}_{solv}^B+\varDelta {G}_{solv}^{AB}\\ {}\kern5em =\varDelta Hgas-T\varDelta S-\varDelta {G}_{GB SA}^A-\varDelta {G}_{GB SA}^B+\varDelta {G}_{GB SA}^{AB}\\ {}\kern5em =\varDelta Hgas-T\varDelta S+\varDelta \varDelta {G}_{GB}+\varDelta \varDelta {G}_{SA}\\ {}\varDelta {H}_{gas}\approx \varDelta Egas=\varDelta {E}_{intra}+\varDelta {E}_{elec}+\varDelta {E}_{vdw}\\ {}\varDelta \varDelta {G}_{GB}=\varDelta {G}_{GB}^{AB}-\left(\varDelta {G}_{GB}^A+\varDelta {G}_{GB}^B\right)\\ {}\varDelta \varDelta {G}_{SA}=\varDelta {G}_{SA}^{AB}-\left(\varDelta {G}_{SA}^A+\varDelta {G}_{SA}^B\right)\end{array} $$

Where T is the temperature, S is the solute entropy, *ΔGgas* is the interaction energy between A and B in the gas phase, and *ΔG*_*solv*_^*A*^, *ΔG*_*solv*_^*B*^ and *ΔG*_*solv*_^*AB*^ are the solvation free energies of A, B, and AB, which are estimated using a GB surface area (GBSA) method [25, 26]. That is, *ΔG*_*solv*_^*AB*^ = *ΔG*_*GBSA*_^*AB*^ + *G*_*GB*_^*AB*^ + *ΔG*_*SA*_^*AB*^, and so forth. _*ΔGGB*_ and *ΔG*_*SA*_ are the electrostatic and nonpolar terms, respectively. The bond, angle, and torsion energies constitute the intramolecular energy *ΔE*_*intra*_ of the complex, while *ΔE*_*elec*_ and *ΔE*_*vdw*_ represent the receptor-ligand electrostatic and van der Waals interactions, respectively. Because of the constant contribution of − *TΔS* for each complex, we refer to *ΔG*_binding_^∗^ for *ΔG*_binding_ + *TΔS* in the discussion. To verify the quality and validity of the resulting *Os*PsaCD complexes, the relative binding free energy *ΔG*_binding_^∗^ was calculated by using MM-GBSA for post processing snapshots from the MD trajectories. The computational alanine scanning method in MM-GBSA was used to evaluate the important residues. The key residues were mutated to alanine and subsequently the difference in the binding free energies between mutated and wild-type complexes was calculated based on the MM-GBSA approach.

### Electrostatic potential calculations

Electrostatic potential maps were calculated with the Adaptive Poisson-Boltzmann Solver (APBS) [29] according to default parameters (physiological salt concentration of 150 mM, temperature of 298.15 K, solvent dielectric of 78.54, and solute dielectric of 2). Solute charges were distributed onto grid points using a cubic B-spline discretization. The molecular surface was defined by the interface between the radius of a water molecule (1.4 Å), and the solute van der Walls radii.

### Bioinformatics analysis

To perform bioinformatics study, we followed our previous method [30] with minor modifications. The full-length PsaC (gi:743432912) and PsaD (gi:115477831) protein sequences from *Oryza sativa* were used as search queries. We conducted BLASTP [31] searches against the National Center for Biotechnology Information (NCBI) non-redundant database, with a cutoff E-value of 1e-10. To reflect evolutionary diversity of PsaC, we selected the following species (Table 1): four land plants (*Oryza sativa*, *Zea mays, Blossfeldia liliputana,* and *Cuscuta obtusiflora*), one charophyte (*Chara vulgaris*), one pteridophyta (*Huperzia lucidula*), one bryophyte (*Syntrichia ruralis*), one green algae (*Bryopsis hypnoides*), and two strains of cyanobacteria (*Lyngbya sp. PCC 8106*, and *Chamaesiphon minutus*). As to PsaD, the selected species for exhibiting evolutionary diversity were as follows: five land plants (*Oryza sativa, Zea mays, Theobroma cacao, Medicago truncatula,* and *Glycine max*), one bryophyte (*Physcomitrella patens*), two phaeocystis (*Phaeocystis antarctica,* and *Phaeocystis globosa*) and two green algae (*Chlamydomonas reinhardtii,* and *Micromonas pusilla CCMP1545*). To obtain the genes coding for PsaC and PsaD in these species, we used the *OspsaC* and *OspsaD* genes to conduct BLASTN searches against nucleotide databases among these organisms. Gene sequences were aligned using ClustalW [32].

**Table 1:** The distances between residues corresponding to R19_C_ and E103_D_ in *Oryza sativa*

PDB	Species	PsaC	PsaD	Distance (Å)
1jb0	*Synechococcus elongatus*	R18 NH1	E103 OE2	6.6
		R18 NH2	E103 OE1	4.8
2wsc	*Pisum sativum*	R19 NH1	E121 OE2	3.3
		R19 NH2	E121 OE1	6.1
4xk8	*Pisum sativum*	R19 NH1	E175 OE2	6.4
		R19 NH2	E175 OE1	6.1
3lw5	*Pisum sativum*	R19 NH1	E175 OE1	3
		R19 NH2	E175 OE2	5.9
4rku	*Pisum sativum*	R19 NH1	E176 OE1	7.3
		R19 NH2	E176 OE2	6.1
2o01	*Spinacia oleracea*	R19 NH1	E121 OE2	2.6
		R19 NH2	E121 OE1	4.5
4l6v	*Synechocystis sp. PCC 6803*	R19 NH1	E106 OE2	5.5
		R19 NH2	E106 OE1	5.9

### Construction of the phylogenetic tree

To construct phylogenetic tree, we followed the method of Wang [33] with minor modifications. Briefly, protein sequences were aligned using **MU**ltiple **S**equence **C**omparison by **L**og- **E**xpectation (MUSCLE) [34] and the resulting protein alignment was subjected to manual inspection in MEGA6 [35]. Phylogenetic analyses were conducted by Neighbor-Joining (NJ) [36]. The NJ phylogenetic tree was reconstructed using protein Poisson distances [37] and the pairwise-deletion of gap sites implemented in MEGA6 and was evaluated with 1000 bootstrap replicates [38].

### Coarse Grained representation

This atomistic model was converted to coarse grained (CG) model using the protocol described by Marrink [39]. The MARTINI forcefield [40, 41] was used for the CG-MD simulations. Briefly, the non-H atoms were mapping to the CG standard particles and CG ring particles by 4:1 and 2–3:1 mapping schemes. Eighteen CG particle types were defined and divided into four main categories: polar (P), charged (Q), mixed polar/apolar (N) and hydrophobic apolar (C). The Lennard Jones interactions were used for the inter-particle interactions, including 10 subtypes to reflect the hydrogen bonding capabilities or polarity.

The protein backbone was represented by a single particle, while the side chain was described by zero or four particles. The Elastic Network (EN) Modeling was built across the CG backbone beads, aiming to maintain the protein secondary structure. The EN scaffold component was based on the MARTINI molecular force field [40, 41]. Two backbone beads were linked by a spring with force constant and a predefined distance cutoff value. In this study, each monomer possessed its own separate EN scaffold, using the same value of R_C_ and K_SPRING_. This design will let the monomer move independently with respect to the other one during the MD simulation.

### Coarse grained MD simulations

All CG-MD simulations were performed using GROMACS 4.5.3 [17]. The Berendsen weak coupling algorithm was used to maintain the temperature (coupling constant of 0.5 ps; reference temperature 300 K) and pressure (coupling constant of 1.2 ps; reference pressure 1 bar), respectively. The nonbonded interactions were treated with a switch function from 0.0 to 1.2 nm for the Coulomb interactions, and from 0.9 to 1.2 nm for the Lennard-Jones interactions. The integration time-step was set to 20 fs and the neighbor list was updated every 5 steps. Each EN system was solvated in a rectangular box with a minimum of 1.0 nm between any protein bead and the edge of the box. After energy-minimization with position restraints (1000 kJ mol-1 nm-2) applied to all protein beads, a 50 ps MD simulation using a 1 fs time-step with the same position restraints was used to relax both the solvent molecules and the protein in the force field. The system was further relaxed with 1 ns long MD run, using a 20 fs time-step and position restraints on the protein “backbone” beads. Finally each system was simulated 500 ns for production without any restraints. In order to convert the CG protein representation, four steps were used according from the protocol described by Sansom and coworkers [42]. Firstly, the backbone of the protein was grown from the CG Cα particles using the Pulchra algorithm [43]. Secondly, the missing side chain atoms of the protein were added using in MODELLER [44]. Note that the advantage of these two steps is to preserve the original coordinates from the CG particles greatly [42]. Thirdly, the protein structure was energy minimized using the conjugant gradient and steepest descents approach to reduce the internal steric clashes of the model.

## Results

### Stability of AT-MD simulations

**Fig. 1:**
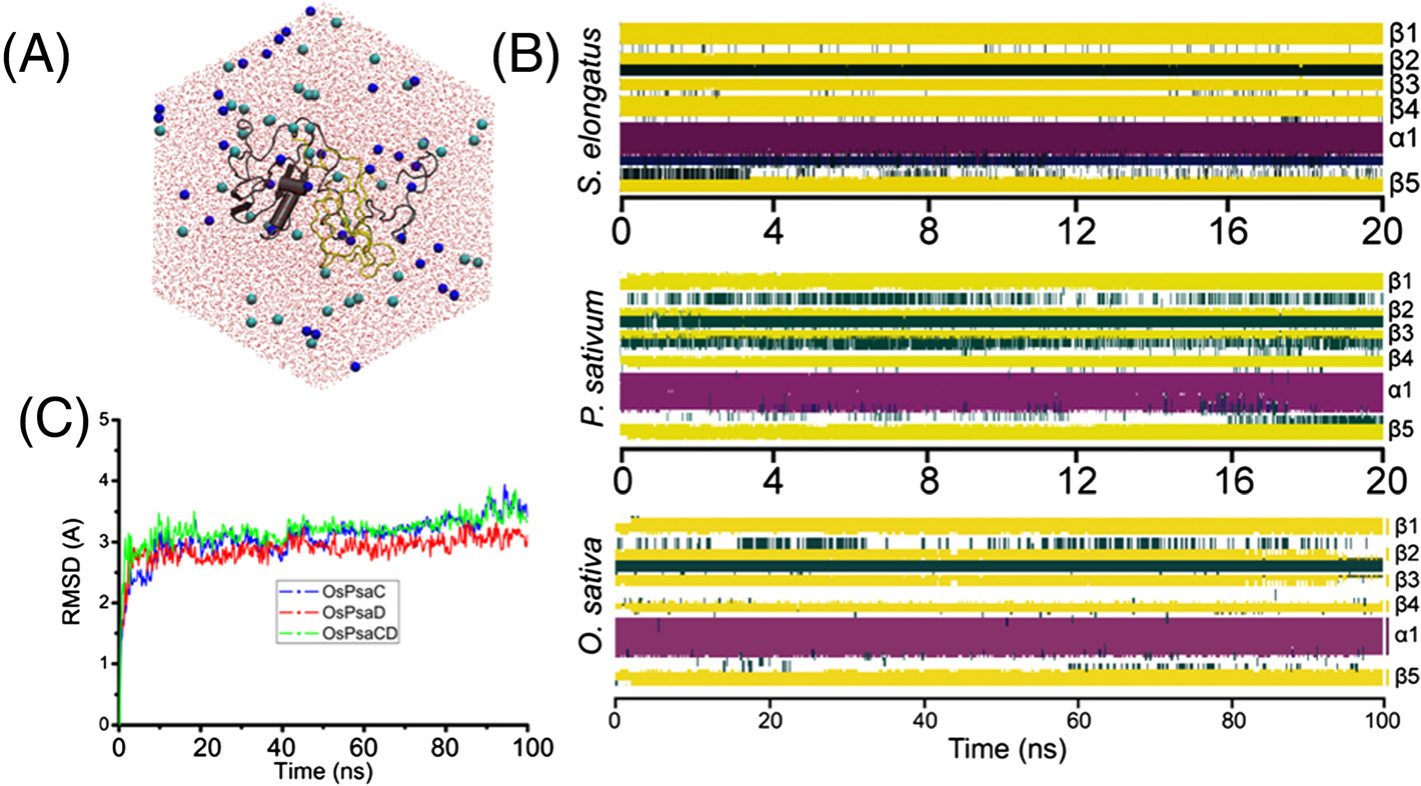
**a** System setup: top view of the model *Os*PsaC-*Os*PsaD complex. *Os*PsaC and *Os*PsaD were solvated in the water solvent. Shown in A was the protein in cartoon representation (*Os*PsaC in yellow and *Os*PsaD in gray), the waters in CPK representation (colored in red), and the ions in balls representation. Rendered with VMD [23]. **b** RMSD of the *Os*PsaC-*Os*PsaD complex with respect to the starting structure for Cα atoms of *Os*PsaC (blue), of *Os*PsaD (red), and of *Os*PsaC-D (green) during the 100 ns production simulation. **c** Time evolution of secondary structure elements in the *S.elongatus*, *P.sativum*, and *O.sativa*, in which β-sheets and α-helices were shown in yellow and red

To access the stability of the *Os*PsaC-*Os*PsaD complex, we monitored its structural drift with respect to the initial conformation just prior to the production MD simulation (Fig. 1a). The Cα RMSD values for *Os*PsaC-*Os*PsaD plateaued around 3 Å during simulations, exhibiting the stability of the protein complex. In addition, we monitored the secondary structure property of *Os*PsaC-*Os*PsaD during simulations, and compared it with that in *S.elongatus* and *P.sativum* (Fig. 1b). Our results suggest *Os*PsaC and *Os*PsaD were able to maintain their structural integrity during most of simulations, exhibiting our model had reached a conformational steady state.

**Fig. 2:**
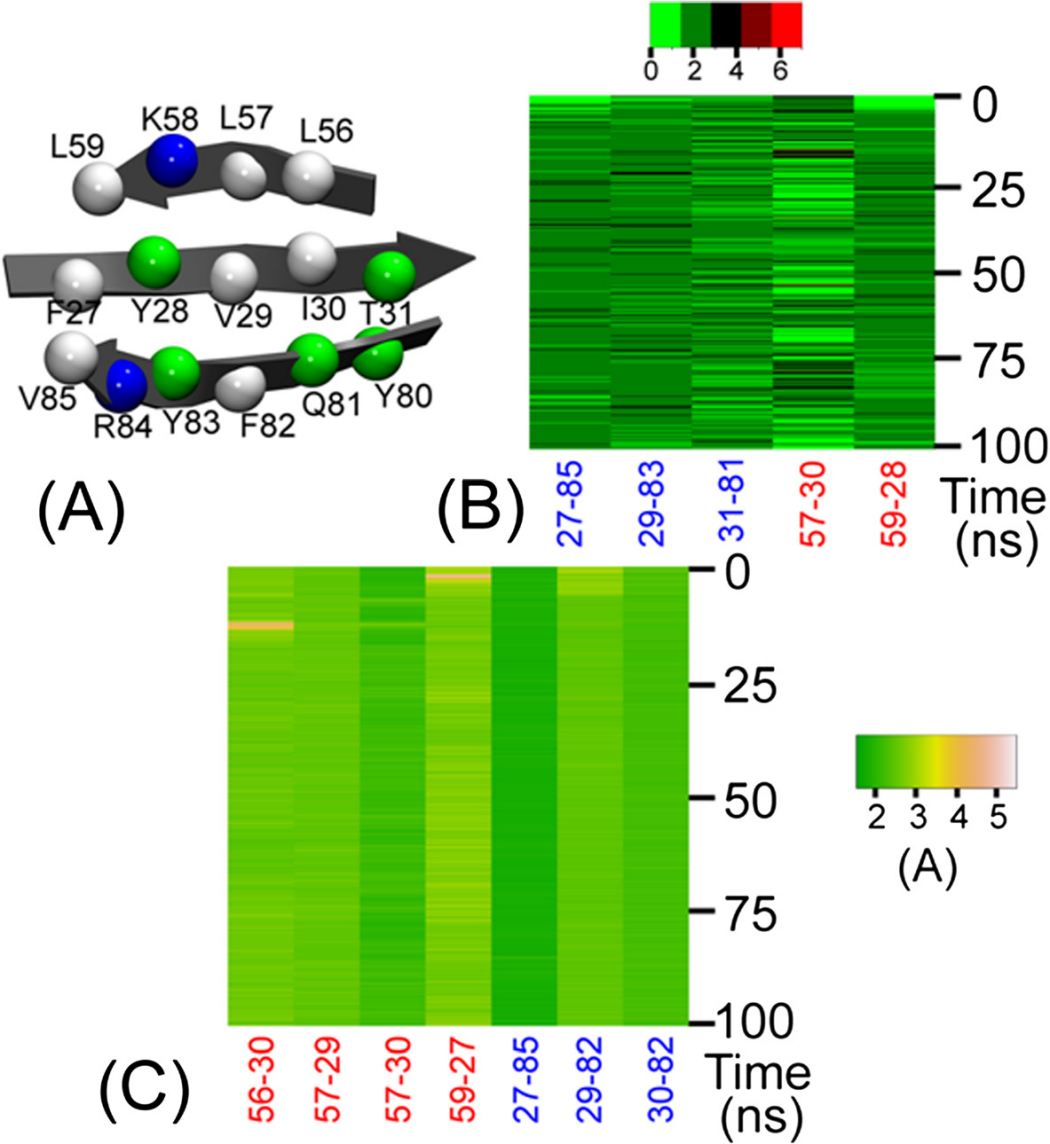
(**a**) Residues maintaining the stability of β-sheets in *Os*PsaD. The number of H-bonds (**b**) in residue pairs F27-V85, V29-Y83, T31-Q81, L57-I30, L59-Y28, and the distance between the closest atoms (**c**) in the residue pairs L56-I30, L57-V29, L57-I30, L59-F27, and F27-V85, V29-F82, I30-F82 during 100 ns MD simulation

Given that β-sheets were widely distributed in the structural architecture of PsaD, we evaluated the stability of β-sheets during the simulations. Fig. 2 suggested both H-binding interactions and hydrophobic interactions played important roles in maintaining β-sheets of *Os*PsaD. Observably, significant hydrogen-bonding interactions stabilizing the β1-β2 and β1-β3 strands were found in the residue pairs F27-V85, V29-Y83, T31-Q81, L57-I30 and L59-Y28 (Fig. 2b). In addition, we measured the hydrophobic interactions by calculating distance between the closest atoms. Fig. 2c exhibited the hydrophobic interactions in the residues pairs L56-I30, L57-V29, L57-I30, L59-F27 of the β1-β2 strand, and F27-V85, V29-F82, I30-F82 of the β1-β3 strand. Hence, the stability of β-sheets also supported our simulation system had reached its equilibrium state.

### H-bonding interactions between OsPsaC and OsPsaD

**Fig. 3:**
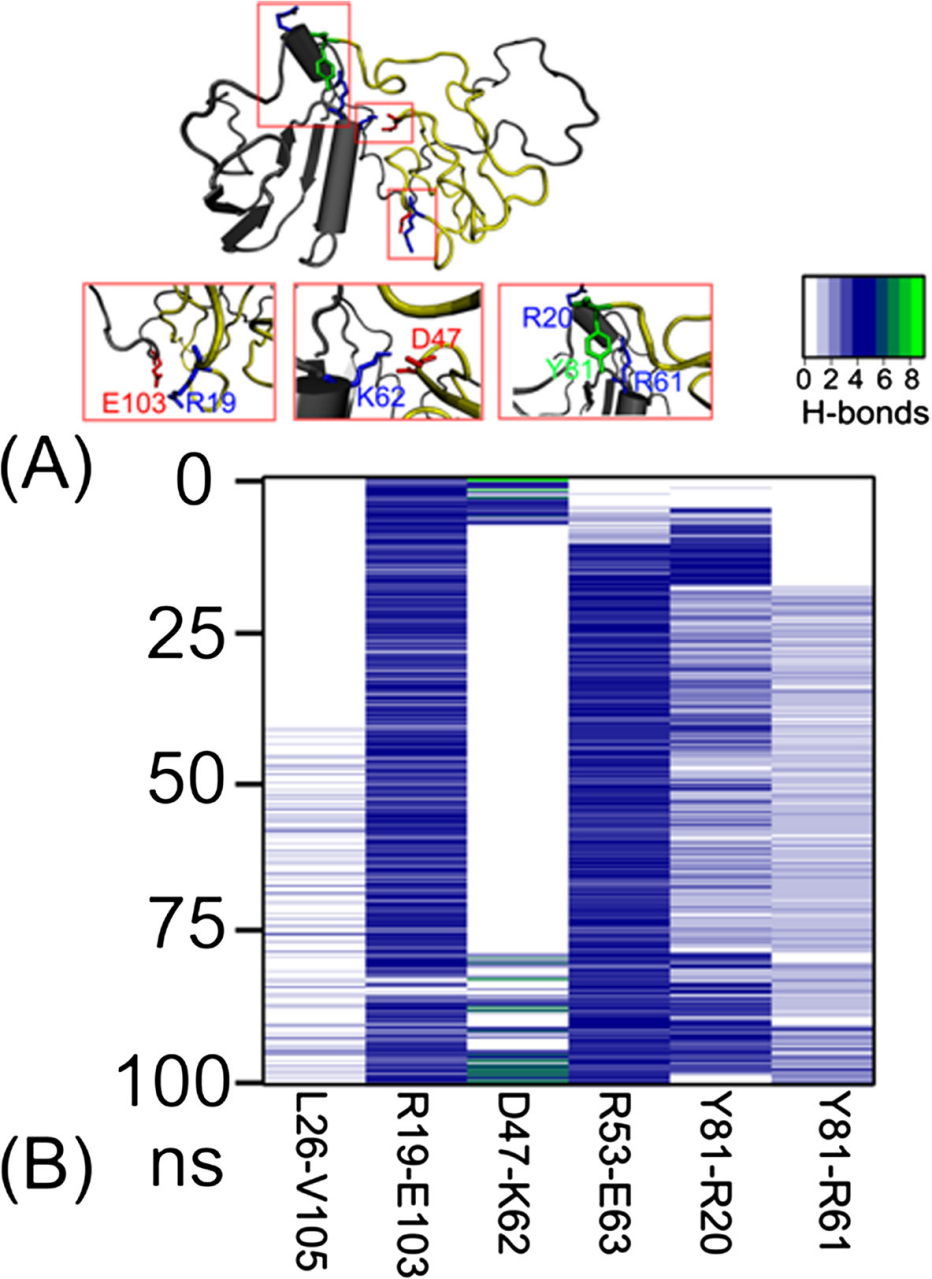
**a** A close view of key residues in the binding of *Os*PsaC to *Os*PsaD. **b** The H-bonds occupancy between the interaction residues during 100 ns MD simulations

Given that the H-bonding interactions play key roles in maintaining protein stability, we analyzed the H-bonding occupancy between *Os*PsaC and *Os*PsaD during MD simulations. Two charged residues R19_C_ and E103_D_ were able to form a salt-bridge, occupying most of the simulation time (Fig. 3a and b). Interestingly, previous study [1] showed the importance of salt bridges in the binding of PsaC to PsaD. Thus, we also calculate the energy contribution of these two residues in the following part. In the neighbor of E103_D,_ V105_D_ formed the hydrophobic interaction with L26_C._ Besides, a salt-bridge existed in the residue pair D47-K62, which was occasionally disrupted in the middle of the simulation. In addition, we also observed some H-bonding interactions in the residue pairs R53-E63, Y81-R20 and Y81-R61. However, these interactions were not pretty stable at the beginning of simulations.

### Computational alanine scanning

**Fig. 4:**
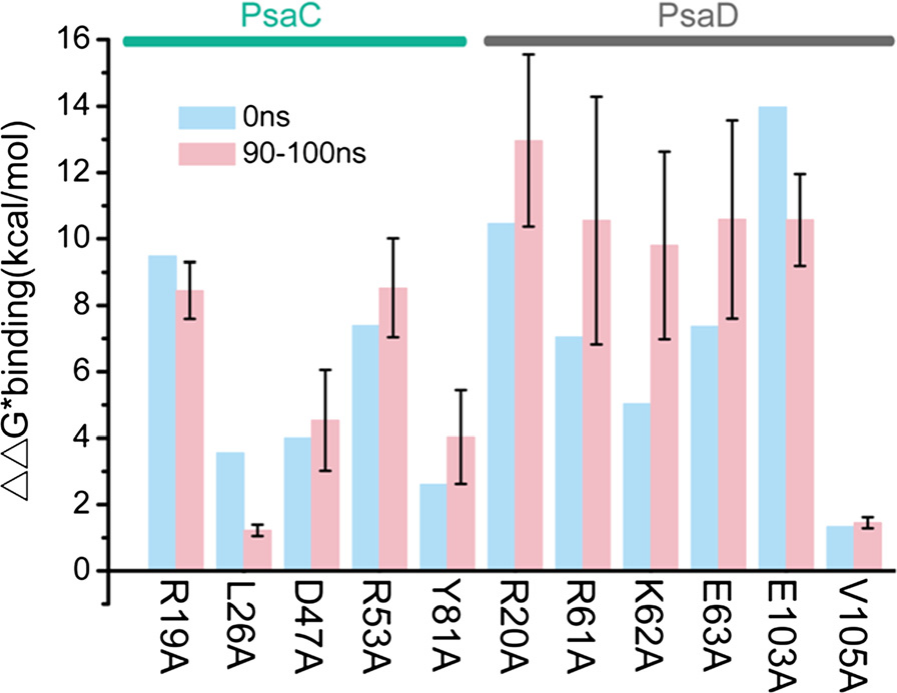
Binding affinities estimated from computational alanine scanning for mutations of the *Os*PsaC-*Os*PsaD complex ((*ΔΔG*_binding_^∗^ = *ΔG*_binding(mutant)_^∗^ − *ΔG*_binding(wild ‐ type)_^∗^). *ΔΔG*_binding_^∗^ results for 11 single mutations in either *Os*PsaC or *Os*PsaD in the initial structure at 0 ns (green) and averaged over 10 structures at the end of the 100 ns simulation (pink; error bars represent standard deviations)

To further explore the recognition residues in the *Os*PsaC-*Os*PsaD complex, we evaluated relative binding free energies by MM-GBSA, and compared interaction energy differences between the mutants and the wild-type by the computational alanine scanning (Fig. 4). We used *in-silico* method to construct mutants following our previous method [10] The residues were mutated to alanine, and subsequently the difference in the binding free energies between mutated and wild-type complexes was calculated based on the MM-GBSA approach. We conducted equilibrium MD simulations on the 11 independent snapshots extracting from the 100 ns simulation time. These 11 snapshots consisted of one snapshot at 0 ns, and ten snapshots from 90 ns to 100 ns.

The R19A and E103A mutants induced a loss of 9.5 and 14 kcal/mol in *ΔΔG*_binding_^∗^ scanning results in the initial structure (Fig. 4). Importance of these two residues was further supported by the values of 8.5 and 10.6 kcal/mol in the *ΔΔG*_binding_^∗^ scanning over the last 10 ns structures. These results showed a good agreement with previous H-bonding analysis, suggesting they significantly contributed to recognition between *Os*PsaC and *Os*PsaD. Besides, two charged residues D47_C_ and K62_D_ lost the energy 4.0 and 5.0 kcal/mol respectively in the initial structure (Fig. 4), and 4.5, 9.8 kcal/mol over the last 10 ns snapshots when they mutated to the alanine, suggesting their importance for the recognition affinity. Also, we noticed three residues R20_D_, R61_D_ and Y81_C_ corresponded to the loss of energy 10.4, 7.0, 2.6 kcal/mol in the initial structure, and 12.9, 10.5, 4.0 kcal/mol over the last 10 ns snapshots.

### CG-MD simulations

**Fig. 5:**
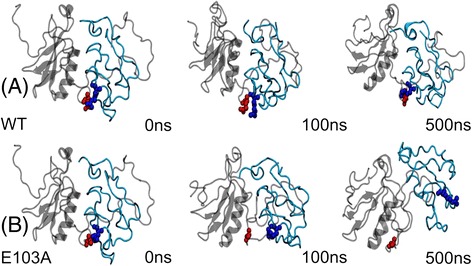
Snapshots at 0 ns, 100 ns and 500 ns were extracted from 500 ns CG-MD simulations for WT (**a**) and E103A mutant (**b**)

To further investigate the effect of the E103A mutation on *Os*PsaC-*Os*PsaD, two 500 ns CG-MD simulations were conducted for the wild-type (WT) system and the E103A mutant (Fig. 5). Expectantly, the salt bridge R19-E103 was able to stabilize in the WT system during the whole 500 ns simulation. This is consistent with previous results from the AT-MD simulations. Significantly, we found domain rearrangements of *Os*PsaC in the E103A mutant. At the beginning of CG-MD simulation, R19 and A103 can still bind tightly. However, these two subunits deviated at around 100 ns due to the disruption of their H-bonding interactions. At 500 ns, the R19-A103 interaction was thoroughly disrupted and conformational change can be observed in *Os*PsaC. Results from CG-MD simulations verified the importance of the salt bridge R19-E103 to maintain the *Os*PsaC and *Os*PsaD interaction.

### Electrostatic potentials maps

**Fig. 6:**
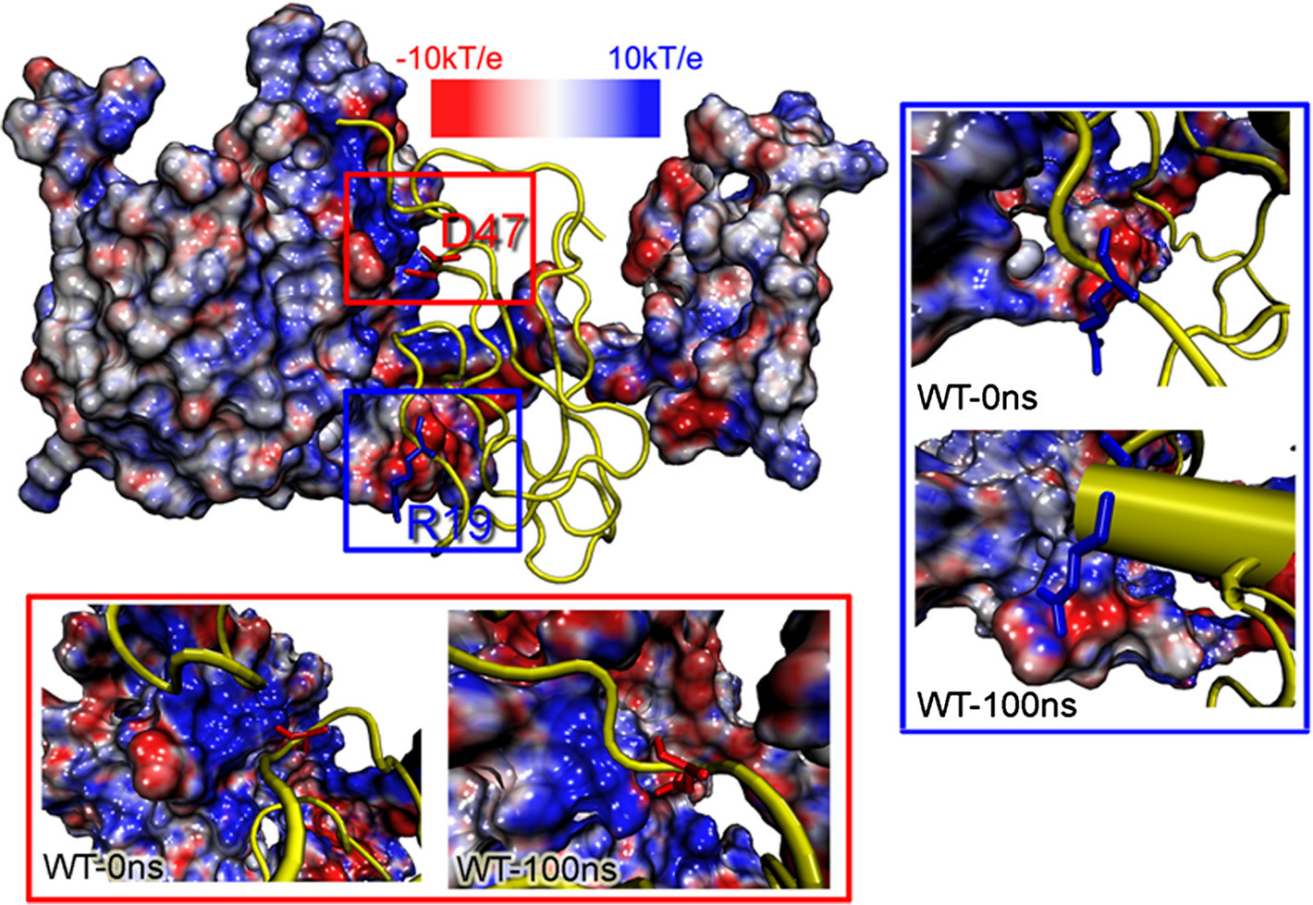
Skin representation of the molecular electrostatic potential surface (EPS) of *Os*PsaD at 0 ns and 100 ns according to the electrostatic potentials. Red: ESP < −10 kT/e, blue: > + 10 kcal/e, grey: between −10 and +10 kT/e

The Electrostatic Potential Surfaces (EPS) of PsaD was claculated with Linearized Poisson-Boltzmann Equation (LPBE) mode in the APBS package. This method solves a molecule using an algorithm of single DH sphere boundary condition and generates a 3D surface skin model colored by electrostatic potential. Dielectric constants for protein and solvent were 2.0 and 78.0 in the calculations. We extracted *Os*PsaC-*Os*PsaD structures at 0 ns and 100 ns. Fig. 6 showed R19 with positive charge was embraced by negative electrostatic potentials. Similarly, the negative charged residue D47 was in the neighbor of electropositive potentials. Apparently, electrostatic attraction plays an important role in the process of *Os*PsaC binding to *Os*PsaD, which is consistent with previous energy analysis.

### Bioinformatics analysis

**Fig. 7:**
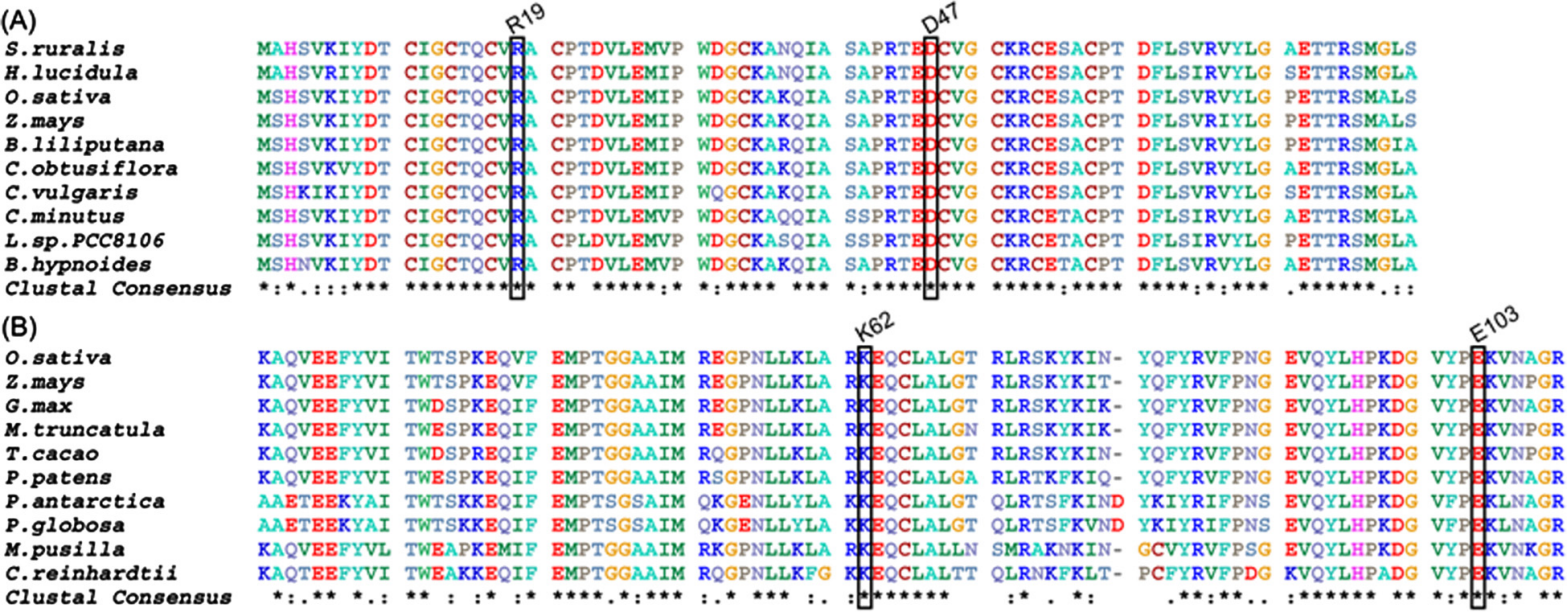
Sequence alignments of PsaC ? (**a**) and PsaD (**b**) in different organisms. Four residues R19, D47, K62 and E103 were highlighted

To investigate if the recognition mechanism between *Os*PsaC and *Os*PsaD is a general mechanism in other organisms, we conduct amino acid homology search and sequence alignments. Using BLASTP to search protein databases in NCBI, we found the protein sequence of *Os*PsaC had a high similarity to the PsaC proteins in land plants. Figure 7 showed R19 and D47 of *Os*PsaC were identical to those in other organisms such as *Z.mays, B.liliputana, C.obtusiflora, S.ruralis, B.hypnoides, C.vulgaris, H.lucidula, C.minutus,* and *L.sp.PCC8106.* Table S1 exhibited *Os*PsaC shares a similarity of 99 % identity to *Zm*PsaC from *Z.mays,* 95 % identity to *Bl*PsaC from *B.liliputana.* The gene sequence of *Os*PsaC shares a similarity of 97 % identity to *Zm*PsaC from *Z.mays,* 89 % identity to *Bl*PsaC from *B.liliputana* (Additional file 1: Table S2 and Figure S3)*.* Supportively, the phylogenetic analysis exhibited *Os*PsaC can be clustered together with *Zm*PsaC and *Bl*PsaC (Additional file 1: Figure S1). Besides, Table S2 showed the gene sequence of *OspsaC* shares a similarity of 79 % identity to *CmpsaC* from *C.minutes and* 76 % identity to *LspsaC* from *L.sp. PCC 8106.* This is mainly because *C.minutes* and *L.sp. PCC 8106* are cyanbacterias, and land plants PsaC and cyanbacteria PsaC are with a different evolutionary origin (Additional file 1: Figure S1).

Figure 7 also showed E103 and K62 of *Os*PsaD were present in all nine other organisms: *G.max, Z.mays, T.cacao, P.patens, M.pusilla, M.truncatula, C.reinhardtii, P.antarctica,*and *P.globosa.* Significantly, Table S1 exhibited the protein sequence of *Os*PsaD has a high similarity to the PsaD proteins in land plants. For instance, it shares a similarity of 82 % identity to *Zm*PsaD from *Zea mays.* The phylogenetic analysis showed *Os*PsaD was clustered together with *Zm*PsaD (Additional file 1: Figure S2), emphasizing the land plants share an evolutionary origin. In addition, we found *Os*PsaD was not clustered with *Pa*PsaD from *P.antarctica* and *Pg*PsaD from *P.globosa*. Supportively, the gene sequence of *OspsaD* only shares 32 % identity to *PapsaD* and 54 % identity to *PgpsaD* (Additional file 1: Table S2 and Figure S4)*.* Besides, *Os*PsaD was not clustered with *Cr*PsaD from *C.reinhardtii* and *Mp*PsaD from *M.pusilla CCMP1545* (Additional file 1: Figure S2). Thus, *Os*PsaD may have a different evolutionary origin from *Pa*PsaD, *Pg*PsaD, *Cr*PsaD and *Mp*PsaD.

### Comparison of X-ray structures

**Fig. 8:**
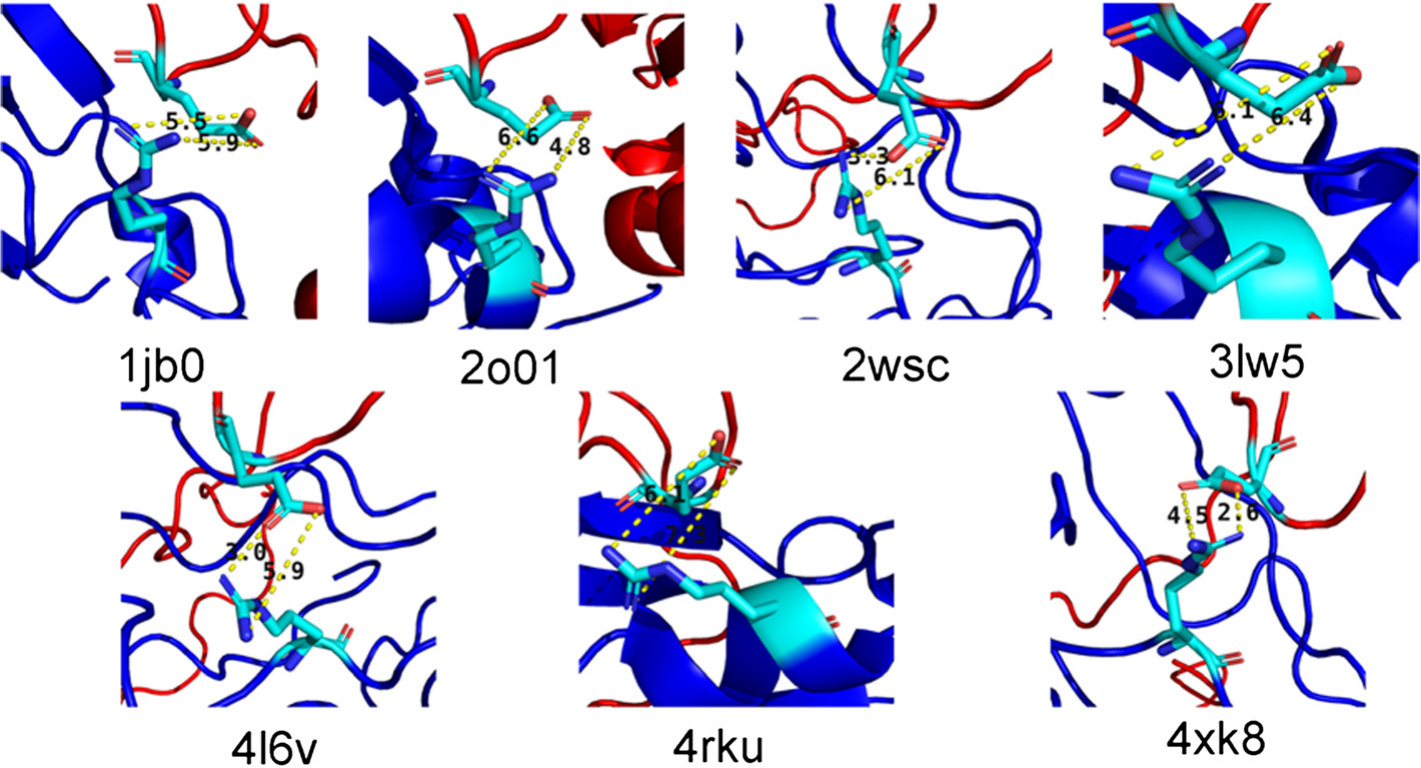
Skin representation of the EPS of PsaD extracted from the X-ray structures. Red: ESP < −5 kT/e, blue: > + 5 kcal/e, grey: between −5 and +5 kT/e. Residues corresponding to R19 and D47 of OsPsaC were shown in their mesh surfaces (R19 is shown in green and D47 is shown in magentas)

By searching the whole Protein Data Bank (http://www.rcsb.org/), we extracted seven different PsaC-PsaD complexes whose three-dimensional structures were known by X-ray technique. These seven structures were from four different organisms: *Synechococcus elongatus* (PDB: 1jb0), *Pisum sativum* (PDB: 2wsc, 4xk8, 3lw5, 4rku), *Spinacia oleracea* (PDB: 2o01) and *Synechocystis sp. PCC 6803* (PDB: 4l6v). We calculated the EPS of PsaD using the APBS package. Figure 8 suggested the D47 binding region of PsaD was strongly electropositive. This region was widely distributed and had a tendency to form a positive pocket. Besides, the region of PsaD facing R19 of PsaC was electronegative, which was partially embraced by its neighboring electropositive region. These observations showed the charge distribution on the surface of PsaD played important roles for orienting residues E47 and R19 of PsaC, and the proper interactions between PsaC and PsaD was initiated by electrostatic forces.

**Fig. 9:**
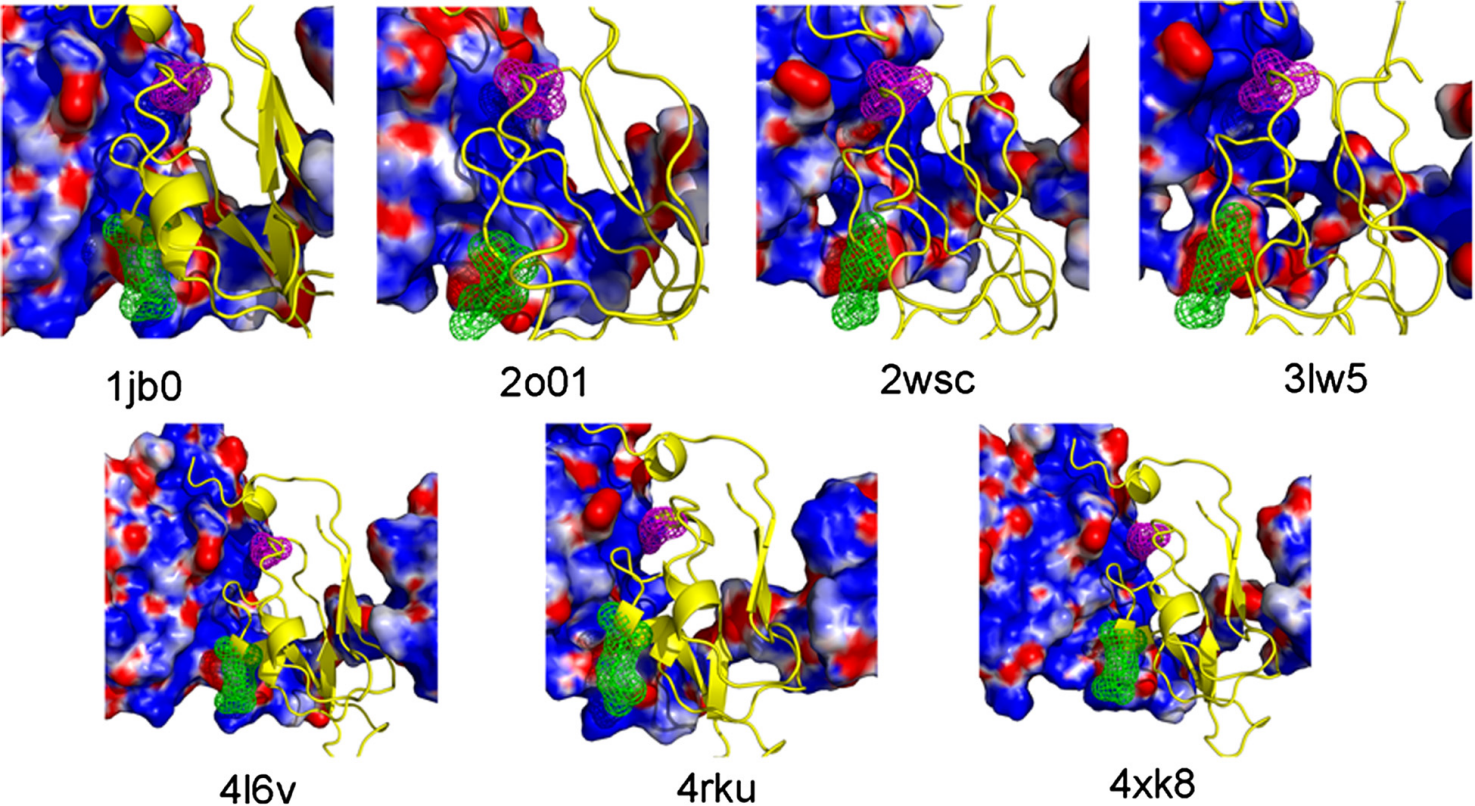
A close view of the salt bridge between R19_C_ and E103_D_ in different X-ray structures. PsaC was shown in blue and PsaD was shown in red. Residues corresponding to R19_C_ and E103_D_ in *Orysa sativa* were shown in their stick representation

Besides, we also examined the salt bridge of R19-E103 in other X-ray structures, the formation of which is thought to be the main reason to stabilize the *Os*PsaC-*Os*PsaD complex. Figure 9 and Table 1 showed the NH1 and NH2 atoms of R19 were juxtaposed against the OE1 and OE2 atoms of E103, suggesting the salt bridge of R19-E103 widely existed in other organisms. Particularly in two higher plants *Pisum sativum* and *Spinacia oleracea*, the atomic distances between NH1 of arginine and OE2 of glutamic acid were 3.3 and 2.6 Å, which were similar with that in *Orysa sativa*. Interestingly, in two blue-green alagas *Synechocystis sp. PCC 6803* and *Synechococcus elongatus*, the residual distances between arginine and glutamic acid were a little far. We noticed the atomic distances between NH2 of arginine and OE1 of glutamic acid were 5.9 and 4.8 Å, indicating evolutionary diversity between higher plants and alagas.

### Validation of the MD model by protein docking

Given that our findings based on computational simulations, it is therefore necessary to apply the complementary method to validate these results. To this end, we performed the protein docking experiment using ClusPro 2.0 [31, 45–47] due to its success in the CAPRI (Critical Assessment of Predicted Interaction). We used *Os*PsaD and *Os*PsaC as receptor and ligand for docking. Free energy scores of each cluster were used to screen candidates because they can provide good hints according to our previous work [10]. Significantly, we found two docking models exhibiting better scores. The scores for the docking model 1 and 2 were −723.9 and −824.9 kcal/mol, suggesting they were favorable binding modes. After superimposing the MD model and two docking models, we found these three models were able to fit well with each other (Additional file 1: Figure S5) and the C-terminal loop of PsaD can form a long arm to interact with PsaC. Taking a closer look at residues R19_C_, D47_C_, K62_D_ and E103_D_, we observed two salt bridges R19–E103 and D47-K62 at the PsaC-PsaD interfaces (Additional file 1: Figure S5). This stressed the importance of these two salt bridges in maintaining the interaction between PsaC and PsaD.

## Discussion

A 2011 proteomic experiment [13] showed the stromal ridge complex of rice existed both in its PSI core complex and PSI super complex. Subsequently, this complex was modeled and refined by 15 ns MD simulation. Due to time limitation of simulations, the dynamics behaviors of the stromal ridge complex were still poorly evaluated. In this study, we combined a 100 ns AT-MD simulation and 500 ns CG-MD simulations to explore possible recognition mechanism between these two proteins. The most significant outcome of this study is that we identified two salt bridges R19-E103 and D47-K62, playing key roles to maintain the interaction between *Os*PsaC and *Os*PsaD. H-bonds analysis and free energy calculation suggested they contributed significantly to binding affinity between *Os*PsaC and *Os*PsaD. Specifically, electrostatic potential surfaces exhibited electrostatic attraction function in maintaining the interface of the *Os*PsaC-*Os*PsaD complex. In addition, CG-MD simulations emphasized the importance of salt bridge R19-E103 during the process of *Os*PsaC recognizing *Os*PsaD, the disruption of which would induce a significant conformational change of *Os*PsaC. Sequence alignments indicated the full sequence of *Os*PsaC’s was relatively conservative, and in particular, two residues R19 and D47 of PsaC in O.sativa were 100 % identical to those in other organisms. As to *Os*PsaD, its sequence exhibited little discrepancy in different organisms. For instance, PsaD in *O.sativa, P.patens, P.antarctica* and *P.globosa* lost partial of their N-terminal residues, indicating diversity of photosynthesis systems in different organisms during evolution. Interestingly, residues E103 and K62 of PsaD in *O.sativa* are 100 % identical to those in other organisms, strongly suggesting conservatively of two salt-bridges R19-E103 and D47-K62 in most of the organisms.

This study focused on the recognition mechanism of *Os*PsaC and *Os*PsaD since previous studies [3, 4] suggested the binding of PsaD to PsaC can greatly affect the assembly of the stromal ridge complex. Genetics experiments showed the in-vivo *psaC* deletion mutant lacked the subunit PsaD [4], while the *psaD* deletion mutant still contained PsaC [3]. This suggested PsaD was able to bind PsaC, only after the binding of PsaC to PsaA/B. It should be noted that residues distributing on PsaA/B heterodimer were highly symmetric, allowing PsaC rotate around the pseudo C2 symmetry of PsaA/B. This will lead half probability for the binding of PsaC to PsaA/B in its correct orientation, while also half possibility to bind incorrectly [7]. The binding of PsaD to PsaC is important because it can lock the region of PsaC correctly bound to PsaA/B, which is also supported by our *in silico* data. The binding free energies correlated well with previous structural analysis [1], emphasizing the H-bonding interactions play important roles in *Os*PsaD locking the correctly bound-region of *Os*PsaC and undocking the incorrectly bound-region of *Os*PsaD. Additionally, previous genetics experiments [2] also showed the *psaE* deletion mutant contained both PsaC and PsaD, indicating PsaE is the last stromal ridge protein to bind the PsaA/B-PsaC-PsaD complex and the PsaC-PsaD prerequisite complex is essential for the PsaE assembly.

Our simulations also explained how β-sheets of PsaD maintain their structure integrity and stability. The secondary structure of β-sheets was widely distributed in PsaD. Nuclear magnetic resonance (NMR) experiment [48] showed the free PsaD structure contained 11 % β-sheets. X-ray crystallography [5, 6] showed PsaD contained four β-sheets and one α-helix was connected by two neighboring β-sheets. Thus, it is interesting to understand how β-sheets maintain their stability. Our result stressed both H-bonding and hydrophobic interactions were essential to stabilize β-sheets, indicating the plausible mechanism of general distribution of β-sheets [5, 6, 48].

## Conclusions

The correctly binding of PsaC to PsaD is a key step to form chloroplast stromal ridge complex, which plays important roles in plant photosynthesis. However, the recognition mechanism and dynamics behaviors of PsaC-PsaD are still obscure. This work aims to identify the key interactions between *Os*PsaC and *Os*PsaD by MD simulations and bioinformatics. Observably, a salt bridge R19-E103 is essential to maintain the stability of *Os*PsaC-*Os*PsaD. Its importance is determined in combination of free energy calculation and electrostatic potentials map, and is subsequently verified by two 500 ns CG-MD simulations.

On the other hand, we are also interested in if the recognition mechanism generally exists in the other organisms. To this end, we conduct amino acid homology search and sequence alignments. The results strongly suggest salt-bridges forming residues are relatively conservative. After comparison of different stromal ridge complexes from previous X-ray structures, we notice electrostatic forces provide proper interactions between PsaC and PsaD. Particularly, residues corresponding to R19_C_ and E103_D_ in rice are also able to form the stable salt bridge. This supports the generality of R19-E103 in other organisms. Collectively, this work will provide dynamics behaviors to understand recognition mechanism of the stromal ridge complex in rice.

## Additional file

Additional file 1: Table S1. Identification of the PsaC and PsaD proteins from the NCBI database in this study. Sequence identity to *Os*PsaC and *Os*PsaD were listed. **Table S2.** Identification of the *psaC* and *psaD* genes from the NCBI database in this study. Sequence identity to *OspsaC* and *OspsaD* were listed. **Figure S1.** Phylogenetic tree of the PsaC proteins. The numbers associated with the branches are bootstrap values. Species names are color-coded as follows: green → land plants, light green → charophyte, blue → pteridophyta, light blue → bryophyta, purple → green algae, and black → cyanobacteria. The tree was deposited in TreeBASE under submission ID 18661. **Figure S2.** Phylogenetic tree of PsaD proteins. The numbers associated with the branches are bootstrap values. Species names are color-coded as follows: green → land plants, light green → bryophyta, black → phaeocystis, and blue → green algae. The tree was deposited in TreeBASE under submission ID 18661. **Figure S3.** Sequences alignments of the psaC genes. The sequences encoding R19 and D47 are highlighted. The color code indicates consistency between pairwise alignments (red: high, yellow: middle, blue: low). **Figure S4.** Sequences alignments of the psaD genes. The sequences encoding K62 and E103 are highlighted. The color code indicates consistency between pairwise alignments (red: high, yellow: middle, blue: low). **Figure S5.** Two docking models are superimposed with the MD model. Two salt bridges R19-E103 and D47-K62 are highlighted in their vdW representations. (DOCX 1149 kb)

## Abbreviations

AT-MD: ATomic Molecular Dynamics
CG-MD: Coarse grained molecular dynamics
Cα: Alpha-Carbon
MD: Molecular dynamics
MM-GBSA: Molecular mechanics generalized born surface area
Os: Oryza sativa
PsaA/B: Subunits A and B of photosystem I
PsaC/D/E: Subunits C, D and E of photosystem I
RMSD: Root mean square distance
WT: Wild type
