# Supplementary material for: Molecular dynamics simulation and bioinformatics study on chloroplast stromal ridge complex from rice (Oryza sativa L.)
Source: BMC Bioinformatics. 2016 Jan 12;17:28. doi: 10.1186/s12859-016-0877-0 (PMC4709881; doi:10.1186/s12859-016-0877-0)

**Table S1: Identification of the PsaC and PsaD proteins from the NCBI database in this study. Sequence identity to *Os*PsaC and *Os*PsaD were listed.**

| **PsaC** |  | **PsaD** |  |
| --- | --- | --- | --- |
| **Species** | **Identity (%)** | **Species** | **Identity (%)** |
| **land plants** |  | **land plants** |  |
| *Zea mays* | 99 | *Glycine max* | 76 |
| *Blossfeldia liliputana* | 95 | *Medicago truncatula* | 73 |
| *Cuscuta obtusiflora* | 81 | *Theobroma cacao* | 74 |
| **Bryophyta** |  | *Zea mays* | 82 |
| *Syntrichia ruralis* | 81 | **Bryophyta** |  |
| **Green algae** |  | *Physcomitrella patens* | 79 |
| *Bryopsis hypnoides* | 81 | **Phaeocystis** |  |
| **Charophyte** |  | *Phaeocystis antarctica* | 70 |
| *Chara vulgaris* | 81 | *Phaeocystis globosa* | 69 |
| **Pteridophyta** |  | [**Green algae**](https://en.wikipedia.org/wiki/Green_algae) |  |
| *Huperzia lucidula* | 81 | *Chlamydomonas reinhardtii* | 63 |
| **Cyanobacteria** |  | *Micromonas pusilla CCMP1545* | 74 |
| *Chamaesiphon minutus* | 81 |  |  |
| *Lyngbya sp. PCC 8106* | 81 |  |  |

**Table S2: Identification of the *psaC* and *psaD* genes from the NCBI database in this study. Sequence identity to *OspsaC* and *OspsaD* were listed.**

| ***psaC*** |  | ***psaD*** |  |
| --- | --- | --- | --- |
| **Species** | **Identity (%)** | **Species** | **Identity (%)** |
| **land plants** |  | **land plants** |  |
| *Zea mays* | 97 | *Glycine max* | 67 |
| *Blossfeldia liliputana* | 89 | *Medicago truncatula* | 72 |
| *Cuscuta obtusiflora* | 84 | *Theobroma cacao* | 69 |
| **Bryophyta** |  | *Zea mays* | 99 |
| *Syntrichia ruralis* | 86 | **Bryophyta** |  |
| **Green algae** |  | *Physcomitrella patens* | 66 |
| *Bryopsis hypnoides* | 81 | **Phaeocystis** |  |
| **Charophyte** |  | *Phaeocystis antarctica* | 32 |
| *Chara vulgaris* | 82 | *Phaeocystis globosa* | 54 |
| **Pteridophyta** |  | [**Green algae**](https://en.wikipedia.org/wiki/Green_algae) |  |
| *Huperzia lucidula* | 83 | *Chlamydomonas reinhardtii* | 64 |
| **Cyanobacteria** |  | *Micromonas pusilla CCMP1545* | 74 |
| *Chamaesiphon minutus* | 79 |  |  |
| *Lyngbya sp. PCC 8106* | 76 |  |  |
|  |  |  |  |

**Figure S1: Phylogenetic tree of the PsaC proteins. The numbers associated with the branches are bootstrap values. Species names are color-coded as follows: green 🡪 land plants, light green🡪 charophyte, blue 🡪 pteridophyta, light blue 🡪 bryophyta, purple 🡪 green algae, and black 🡪 cyanobacteria. The tree was deposited in TreeBASE under submission ID 18661.**

**Oryza sativa**

**Zea mays**

**Blossfeldia liliputana**

**Chara vulgaris**

**Cuscuta obtusiflora**

**Syntrichia ruralis**

**Huperzia lucidula**

**Bryopsis hypnoides**

**Lyngbya sp. PCC 8106**

**Chamaesiphon minutus**

86

94

53

37

45

25

19

0.0100

**Figure S2: Phylogenetic tree of PsaD proteins. The numbers associated with the branches are bootstrap values. Species names are color-coded as follows: green 🡪 land plants, light green🡪 bryophyta, black 🡪 phaeocystis, and blue 🡪** [**green algae**](https://en.wikipedia.org/wiki/Green_algae)**. The tree was deposited in TreeBASE under submission ID 18661.**

**Glycine max**

**Medicago truncatula**

**Theobroma cacao**

**Zea mays**

**Oryza sativa**

**Physcomitrella patens**

**Phaeocystis antarctica**

**Phaeocystis globosa**

**Chlamydomonas reinhardtii**

**Micromonas pusilla CCMP1545**

100

94

91

98

85

54

73

0.050

**Figure S3: Sequences alignments of the psaC genes. The sequences encoding R19 and D47 are highlighted. The color code indicates consistency between pairwise alignments (red: high, yellow: middle, blue: low).**

**R19**

*O.sativa* GGATGCACTCAATGTGTA**CGA**GCTTGTCCAACAGAT
*Z.mays* TTTCAGGCCCTAAATAAA**CGC**GGACACTTAAAAAAT
*B.liliputana* GGGTGCACTCAATGTGTA**CGA**GCTTGTCCTACAGAT
*C.obtusiflora* GGATGTACTCAATGTGTT**CGA**GCCTGCCCTACCGAT
*S.ruralis* GGTTGTACTCAATGTGTA**AGA**GCATGCCCTACAGAT
*B.hypnoides* GGTTGTACTCAATGTGTG**CGA**GCTTGTCCGACGGAT
*C.vulgaris* TCTCAGACCCAAGATACA**CTC**TAACACTTAAAAAGT
*H.lucidula* GGTTGTACCCAGTGCGTA**AGA**GCTTGTCCTACCGAT
*C.minutus* TTTCCGCGCCTAAATAAA**CGC**GGATACTCAGGAAAT
*L.sp. PCC 8106* TTTCAGGTCCGAGATAGA**CCC**GAATGCTCAAGAAAT
*cons*          *                         *

**D47**

*O.sativa* AAGCAAATTGCTTCCGCGCCAAGAACCGAA**GAT**TGT

*Z.mays* ACACAATCTTCGGTTCTCGGCGCGGAAGCA**ATT**TGC

*B.liliputana* AGACAAATAGCTTCTGCTCCAAGAACAGAG**GAC**TGT

*C.obtusiflora* AAACAAATTGCCTCAGCTCCAAGAACTGAA**GAC**TGC

*S.ruralis* AATCAAATTGCATCTGCTCCTAGAACAGAA**GAT**TGT

*B.hypnoides* AAACAAATTGCGTCAGCTCCAAGAACAGAA**GAC**TGT

*C.vulgaris* ACACAATCTTCGGTTCTAGGAGCTGAAGCA**ATT**TGT

*H.lucidula* AATCAAATTGCTTCTGCTCCTAGAACAGAA**GAC**TGT

*C.minutus* ACACAATCCTCGGTGCGGGGTGAAGACGCA**ATT**TGT

*L.sp. PCC 8106* ACACAATCTTCTGTACGGGGAGAAGATGCG**ATT**TGA

*cons* *  ***    *                *     **

**Figure S4: Sequences alignments of the psaD genes. The sequences encoding K62 and E103 are highlighted. The color code indicates consistency between pairwise alignments (red: high, yellow: middle, blue: low).**

**K62**

*O.sativa*   GCCAGG**AAG**GAGCAGTGCCTGGCCCTGGGCACCAGGCTCCGCTCC-AA-GTACAAGATCA
G.max        GCGAGG**AAG**GAGCAGTGCTTGGCTCTTGGGACTAGGCTCAGGTCA-AA-GTACAAGATCA
Z.mays      GCGCGC**AAG**GAGCAGTGCCTGGCGCTCGGCACCAGGTTGCGCTCC-AA-GTACAAGATCA
T. cacao      GCCAGG**AAA**GAGCAGTGCTTGGCGCTCGGTACCAGACTGAGGTCT-AA-GTACAAGATCA
P. patens GCCAGG**AAG**GAGCAGTGTCTTGCACTCGGCGCCAGGCTGAGGACT-AA-GTTCAAAATTC
M. pusilla  GCCCGC**AAG**GAGCAGTGCCTCGCGCTTCTCAACTCCATGAGGGCC-AA-GAACAAGATCA
M. truncatula  GCAAGG**AAA**GAACAGTGTTTGGCTTTAGGAAATAGATTAAGGTCT-AA-GTACAAGATTA
C. reinhardtii GGCAAG**AAG**GAGCAGTGCCTCGCCCTGACGACCCAGCTCCGCAAC-AA-GTTCAAGCTGA
P.antarctica TGTACG**AAG**T-TGAGTGCCTAATGCTAAA-------CATTGTTCTTTTTTTGCAAGGTAA
P.globosa  GCAAAA**AAA**GAACAATGTTTAGCATTAGGCACTCAACTTCGTACA-TCATTTAAAGTTAA
cons                **     * **  *     *              *            **  *

**E103**

*O.sativa*   CG**GAG**AAGGTCAACGCCGGCAGGCAGGGCGTCGGCCAGAACTTCCGCAGCATCGG
G.max        CT**GAG**AAGGTCAACGCCGGACGCCAAGGGGTTGGTCAGAACTTCAGGTCTATTGG
Z.mays      CG**GAG**AAGGTCAACCCCGGCAGGGAGGGCGTCGGCCAGAACTTCCGCAGCATCGG
T. cacao      CT**GAG**AAAGTGAACCCTGGTCGTCAAGGAGTGGGGCAGAACTTCAGATCAATTGG
P. patens CA**GAG**AAGGTGAACGCTGGAAGATCACCCGTCGGAGTGAACAACAGGAGCATTGG
M. pusilla  CC**GAA**AAGGTCAACAAAGGACGCGTCGGGGCCAACAACAACATGCGCTCCATCGG
M. truncatula  CT**GAG**AAGGTTAACCCTGGTCGTCAAGGGGTTGGTCAGAATTTCAGGTCTATTGG
C. reinhardtii CC**GAG**AAGGTGAACGCTGGCCGCGTGGGCGCGAACCAGAACATGCGCCGCATCGG
P.antarctica TT**GTC**CAAGTGATCGCGTA----TTTTTCCTCAGTTTCTGCTGCTCGTAACCAAC
P.globosa  CA**GAA**AAATTAAATGCTGGGCGACTTGGTGTAGGTAATGTTATGCATTCGATTGG
cons            *   *  * *

**Figure S5: Two docking models are superimposed with the MD model. Two salt bridges R19-E103 and D47-K62 are highlighted in their vdW representations.**


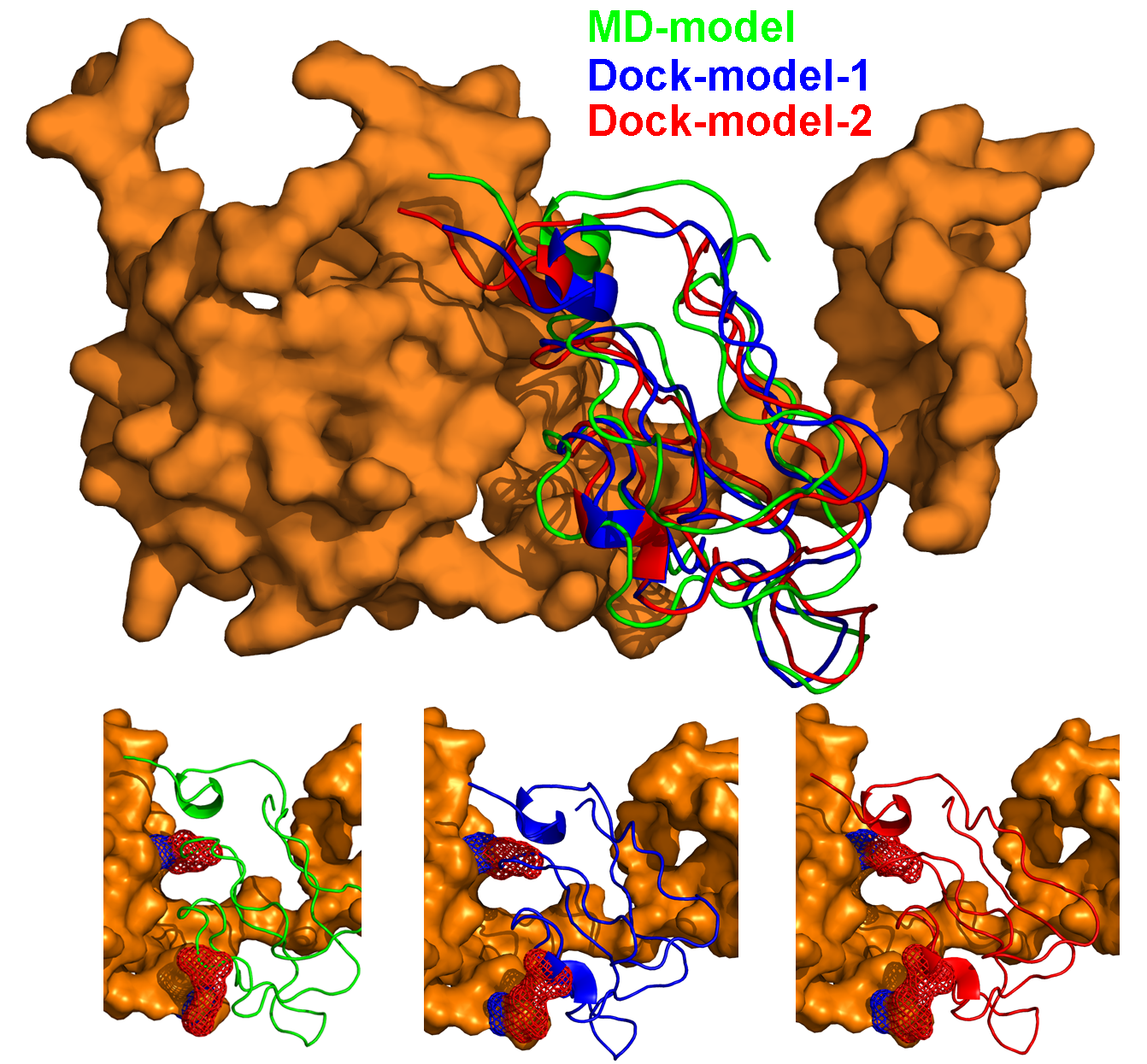

Supplement: Additional file 1: Table S1. — Identification of the PsaC and PsaD proteins from the NCBI database in this study. Sequence identity to OsPsaC and OsPsaD were listed. Table S2. Identification of the psaC and psaD genes from the NCBI database in this study. Sequence identity to OspsaC and OspsaD were listed. Figure S1. Phylogenetic tree of the PsaC proteins. The numbers associated with the branches are bootstrap values. Species names are color-coded as follows: green → land plants, light green → charophyte, blue → pteridophyta, light blue → bryophyta, purple → green algae, and black → cyanobacteria. The tree was deposited in TreeBASE under submission ID 18661. Figure S2. Phylogenetic tree of PsaD proteins. The numbers associated with the branches are bootstrap values. Species names are color-coded as follows: green → land plants, light green → bryophyta, black → phaeocystis, and blue → green algae. The tree was deposited in TreeBASE under submission ID 18661. Figure S3. Sequences alignments of the psaC genes. The sequences encoding R19 and D47 are highlighted. The color code indicates consistency between pairwise alignments (red: high, yellow: middle, blue: low). Figure S4. Sequences alignments of the psaD genes. The sequences encoding K62 and E103 are highlighted. The color code indicates consistency between pairwise alignments (red: high, yellow: middle, blue: low). Figure S5. Two docking models are superimposed with the MD model. Two salt bridges R19-E103 and D47-K62 are highlighted in their vdW representations. (DOCX 1149 kb) [file 12859_2016_877_MOESM1_ESM.docx]
